# Evaluation of Natural Radioactivity Level in Surface Soil from Bambasi District in Benishangul Gumuz Region, Ethiopia

**DOI:** 10.1155/2024/6633673

**Published:** 2024-09-18

**Authors:** Yared Birhane Kidane, Tilahun Tesfaye Deressu, Guadie Degu Belete

**Affiliations:** ^1^ Department of Physics Natural and Computational Science College Addis Ababa University, Addis Ababa, Ethiopia; ^2^ Department of Physics Natural and Computational Science College Assosa University, Assosa, Ethiopia

## Abstract

The study assessed the concentration of natural radionuclides in soil samples from the Bambasi district in Ethiopia's Benishangul Gumuz region using a gamma-ray spectrometer equipped with a high-purity germanium (HPGe) detector. The measured activity concentrations of ^238^U, ^232^Th, and ^40^K in soil samples varied from 46.2 ± 2.25 to 88.49 ± 5.73 Bq/kg, 73.4 ± 4.12 to 119.65 ± 8.45 Bq/kg, and 176.78 ± 8.63 to 396.71 ± 25.39 Bq/kg, respectively. The average concentration of ^238^U and ^232^Th exceeded the recommended worldwide population weighted average values of 32.0 and 45.0 Bq/kg, respectively, while the average concentration of ^40^K was below the recommended value of 420.0 Bq/kg. The mean absorbed dose rate was calculated to be 91.6 ± 5.1 nGy/h, which is above the recommended safe value of 59 nGy/h. The average annual effective dose equivalents for indoor and outdoor exposure were determined to be 2.65 ± 0.14 mSv/y and 0.66 ± 0.1 mSv/y, respectively. The calculated mean values of the internal hazard index, external hazard index, and gamma index across all soil samples were 0.72 ± 0.05, 0.55 ± 0.02, and 0.72 ± 0.02, respectively, all below the recommended safe threshold of one. These findings suggest that the activity concentrations observed in the soil samples exceed safe levels, indicating the necessity for further investigation into radioactivity levels and epidemiological studies regarding potential hazards from high background radiation.

## 1. Introduction

The natural environment has long been imbued with radiation, originating from a myriad of natural and man-made sources. These sources collectively contribute to what we commonly refer to as background radiation, a ubiquitous presence of low-level ionizing radiation constantly surrounding us [1]. Human interaction with this radiation is inherent to our existence, as we ingest or inhale nuclides present in the air, food, and water. Exposure levels vary widely based on factors such as climate, local geology, and human activity patterns unique to each region [2].

One significant source of background radiation stems from radioactive elements naturally present in the Earth's crust, such as uranium, thorium, and their decay products. These elements emit radiation continuously, with radionuclides finding their way into rocks, soil, and building materials, particularly in regions with elevated concentrations of these elements. This study focuses on analyzing soil radioactivity in samples collected from the Bambasi area in western Ethiopia [2, 3].

Soil, being a complex matrix, hosts a diverse array of both naturally occurring and anthropogenic radionuclides. Monitoring soil radioactivity is of paramount environmental concern due to its potential impacts on human health and ecosystems. The concentration and distribution of radionuclides in surface soils are crucial factors in assessing environmental radioactivity levels, evaluating associated health risks, and formulating effective regulatory measures [2, 4]. Radionuclides present in soil emit gamma radiation, contributing to alterations in background radiation levels in the environment. The extent of this radiation depends on various geographical and geological factors. Radionuclides such as thorium and uranium may undergo redistribution during geological evolution cycles, resulting in small concentration deposits under favourable geological processes [1, 5].

Zircon, a mineral found in nature, serves as a source of uranium and thorium and occurs in sedimentary, igneous, and metamorphic rocks. Commonly, zircon along with thorite is a source of Th, U, Y, and heavy rare earth elements (HREE) [6]. The mobilization of radionuclides from rock to soil is influenced by specific concentrations, indicating leaching of ^238^U to rock rather than soil, lower mobility of thorium from rock to soil due to its stability, and a higher concentration of potassium (^40^K) in soil compared to rocks, as potassium is a mobile element [7].

Everyone in the world is exposed to; in fact lives with these background radiation levels, with external exposure occurring through irradiation and internal exposure resulting from ingestion and inhalation. The predominant source of radiation exposure for humans is the natural environment, which contributes up to 85% of the annual exposure dose received globally [1, 8]. Soil, a foundational component of our environment, is comprised of a complex mixture of mineral particles, organic matter, air, and water. Its composition and structure vary significantly across different regions, influencing the types of vegetation it can support [9].

Understanding how radionuclides interact with soil and plants is crucial, as plants can absorb these radionuclides and transport them to edible parts. This absorption process depends on factors such as the radionuclide's chemical availability and its proximity to the root zone [10]. The primary aim of this study was to determine the concentration of naturally occurring radionuclides in various soil samples collected from the Bambasi district in the Benishangul Gumuz region, Ethiopia. This research aimed to categorize the radioactivity concentration of soil samples as either safe or hazardous by comparison of the results with the recommended safe values set by the United Nations Scientific Committee on the Effects of Atomic Radiation (UNSCEAR).

## 2. Materials and Methods

### 2.1. Description of the Study Area

**Figure 1:**
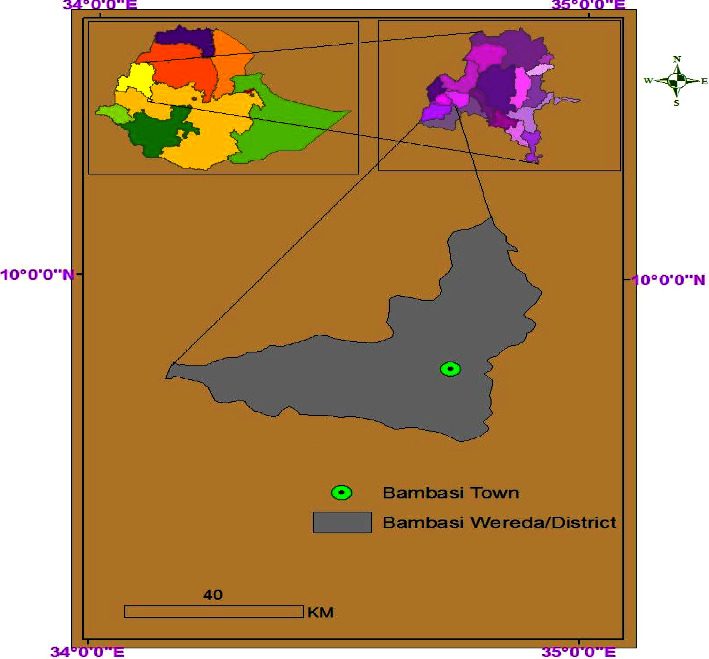
Location map of Bambasi district using ArcGIS 10.2.

Benishangul Gumuz region is one of the eleven regional states comprising the Ethiopian federal structure. Bambasi woreda is situated in Ethiopia, approximately 614 km away from the capital city, Addis Ababa (Figure 1). Its geographical coordinates are within latitude 9°–10° N and longitude 034°–035°E [11]. Bambasi district is located in the Assosa zone. Assosa zone is one of the three zones situated in the Benishangul Gumuz Regional State (BGRS). Assosa zone is mainly known for its potential gold deposits, holding promise for high-profit opportunities [12].

### 2.2. Sample Collection and Preparation

Samples were obtained from specific locations in Bambasi town. Bambasi district chosen in this study was based on, increasing of its population density, having a high agricultural demand, and there are gold mining activities. A total of eight sites in Bambasi town, as shown in Table 1, were selected for the collection of surface soil samples. Each sample was carefully dug out from a depth of 5–30 cm of the surface soil, with an approximate weight of 1 kg per sample. These samples were coded as Bambasi-S1 to Bambasi-S8, and their respective locations are outlined in Table 1.

After collection, the samples were securely packed in polyethylene bags and transported to the laboratory of the Ethiopian Conformity Assessment Enterprise (ECAE) for further processing. The gathered soil samples underwent air-drying at 100°C for 10–24 hours, depending on the soil moisture content. Subsequently, the dried samples were crushed to ensure homogeneity, with the crushing process continuing until the soil samples reached a powder-like consistency. A 0.25 mm mesh was employed for sieving the samples. Around 500 g of the homogenized soil samples were then packed and sealed in airtight cylindrical Marinelli beakers and stored for 28 days (four weeks) to achieve secular equilibrium. Finally, the processed samples were transported to the Ethiopian Technology Authority (ETA) for the use of HPGe detector.

### 2.3. Experimental Setups

The gamma-ray emitting radionuclides concentrations in the soil samples were measured by using an HPGe detector coupled with a multichannel analyzer (MCA). HPGe detector is a suitable semiconductor radiation detector, and it has a high-resolution capacity for photo peaks. The Marinelli beaker sample geometry was 538 G-E. The HPGe detector has 77% relative photopeak efficiency. The detector has a 1.8 keV energy resolution. Multichannel analyzer of 8192 channel performance is connected to computer software of Genie 2000. The voltage of 3499 V was used for the detector. The calibrated radionuclides for the detector were ^60^Co and ^137^Cs, which are the quality standard sources according to ISO 9001. The quality standard used for calibrating the detector was ISO/IEC 17025. The detector is enclosed with a lead shield (100 mm thickness), cadmium (2 mm thickness), and copper (2.5 mm thickness) to prevent the background radiation that comes from the surroundings. The calibration of the experiment was done using IAEA-certified standard reference sources. The counting time of the gamma-ray spectrum was 28,800 s. The soil samples were placed in their Marinelli beakers, directly on the front face of the detector.

The peak areas in the spectrum were calculated, and the background radiation counting was subtracted to obtain the net count rate. The minimum detection limits of the detector according to ISO 11929 reports were 1.33, 1.02, and 4.99 Bq/kg for ^238^U, ^232^Th, and ^40^K, respectively. The specific energy spectrum for each radionuclide was obtained by photopeak area analysis. The specific activity of ^238^U was assessed from gamma-ray lines of ^214^Pb at 351 keV and ^214^Bi at 609.3 and 1764.5 keV, while the activity of ^232^Th was evaluated from gamma-ray lines of ^228^Ac at 338.4, 911.1, and 968.9 keV, ^212^Pb at 238.63 keV, and ^208^Tl at 583.19 keV. The specific activity of ^40^K was directly determined from its gamma-ray line at 1460.8 keV [13].

### 2.4. Measurements of Activity Concentration

The specific activity concentrations (*A*_*c*_) in Bq/kg of the soil samples were calculated as follows [14]:

(1) $$\begin{array}{c} A_{c}=\frac{N_{c}}{I\varepsilon MT_{s}}. \end{array}$$

where *A*_*c*_ is radioactivity concentration of the samples (Bq/kg), *N*_*c*_ is the net count for packed sample (*N*_*s*_) minus count for the background (*N*_*b*_), *ε* is efficiency of the detector for the gamma ray energy of interest, *I* is probability of gamma-ray absolute intensity, *M* is mass of the packed sample (kg), and *T*_*s*_ is the actual sample counting time. The error associated with each measured activity of the samples was calculated using standard deviation (*σ*_*s*_) [15].

(2) $$\begin{array}{c} \text{Count rate}=\frac{N_{c}}{T_{s}}=\frac{N_{s}}{T_{s}}\pm \sigma_{s},\\ \text{where }\sigma_{s}=\sqrt{\frac{N_{t}\;}{{T_{t}}^{2}}+\frac{N_{b}}{{T_{b}}^{2}}}, \end{array}$$

where *σ*_*s*_ is the standard deviation of the measured sample, *N*_*t*_ is the total count, *T*_*t* _ is the total count time, and *T*_*b* _ is the background counts time.

### 2.5. Estimation of Radiological Dose and Hazardous Indices

#### 2.5.1. Absorbed Dose Rate (*D*)

This refers to the rate at which ionizing radiation is absorbed per unit of time. The absorbed dose rate in the air, resulting from the uniform distribution of ^238^U, ^232^Th, and ^40^K radionuclides at a height of 1 meter above the ground surface, was calculated using the following formula [1]:

(3) $$\begin{array}{c} D\left(\frac{\text{nGy}}{h}\right)=0.462A_{U}+0.602A_{Th}+0.0417A_{K}. \end{array}$$

The world average absorbed dose rate thresholds are 59 nGy/h for outdoors and 84 nGy/h for indoors.

#### 2.5.2. Annual Effective Dose Equivalent (AEDE)

The annual effective dose equivalent denotes the biological effect equivalent to the deposition of one joule of radiation energy per kilogram of the human body over the course of a year. To estimate the annual effective dose equivalent in mSv/y, the absorbed dose rate values are utilized, employing a conversion coefficient of 0.7 Sv/Gy for converting absorbed doses in the air to the received effective dose. The calculation also incorporates an indoor occupancy factor of 0.8, reflecting that, on average, the population spends 80% of their time indoors. Additionally, an outdoor occupancy factor of 0.2 is considered, implying that 20% of their time is spent outdoors [1].

It is known that the world's annual effective dose rate due to external radiation is 0.41 mSv/y indoors and 0.07 mSv/y outdoors. For people living in a certain area, the annual effective dose equivalent for outdoor and indoor could be calculated using the following equations [1]:

(4) $$\begin{array}{c} \text{AEDE}{\left(\frac{\text{mSv}}{y}\right)}_{\text{outdoor}}=D\left(\frac{\text{nGy}}{h}\right)\times 24\;h\times 365.2\;d\times 0.2\times 0.7\frac{Sv}{Gy}, \end{array}$$

where 0.7 is the absorbed dose conversion factor and 0.2 is the outdoor occupancy.

(5) $$\begin{array}{c} \text{AEDE}{\left(\frac{\text{mSv}}{y}\right)}_{\text{indoor}}=D\left(\frac{\text{nGy}}{h}\right)\times 24\;h\times 365.2\;d\times 0.8\times 0.7\frac{Sv}{Gy}. \end{array}$$

This is for an indoor occupant.

(6) $$\begin{array}{c} \text{AEDE}\left(\frac{\text{mSv}}{y}\right)\text{indoor}=D\times T\times F\times 0.8,\\ \text{AEDE}\left(\frac{\text{mSv}}{y}\right)\text{indoor}=D\left(\frac{\text{nGy}}{h}\right)\times 7010\;{\text{hy}}^{-1}\times 0.7\;\frac{Sv}{Gy},\\ T\times 0.8=24\;h\times 365.25\;d\times 0.8≅7010\;{\text{hy}}^{-1}, \end{array}$$

where *D* is the calculated dose rate in (nGy/h), *T* is the indoor occupancy time factor within a year (8760 h), and *F* is the conversion factor (0.7 Sv/Gy).

#### 2.5.3. Radium Equivalent (Ra_eq_)

The radium equivalent has been calculated by using a standard value of 370 Bq/kg per a sum of the value of 370 Bq/kg, 259 Bq/kg, and 4810 Bq/kg for ^238^U, ^232^Th, and ^40^K, respectively. The equation for radium equivalent activity (Ra_eq_) would be [14]

(7) $$\begin{array}{c} {\text{Ra}}_{\text{eq}}=A_{U}+1.43A_{\text{Th}}+0.077A_{K}. \end{array}$$

For environmental, soil, and building materials, its value should be less than 370 Bq/kg, and for industries, it can be 370–740 Bq/kg [1, 4, 16].

#### 2.5.4. Gamma Index (*Iγ*)

It is one of the measurements of a radiation hazard that comes from gamma radiation, and the recommended maximum value must be less than one. The gamma index *Iγ* is calculated using the following equation [17]:

(8) $$\begin{array}{c} I_{\gamma }=\frac{A_{U}}{300}+\frac{A_{Th}}{200}+\frac{A_{K}}{3000}. \end{array}$$

Its permissible limit is *I*_*γ*_ = 1 corresponds to an absorbed gamma dose rate of 0.3 mSv/y, implying that materials with *I*_*γ*_ ≥ 1 should be avoided.

#### 2.5.5. External Hazard Index (*H*_*ex*_)

The external hazard index is an expression of external exposure that comes from radioactive nuclides. The external hazard index (*H*_*ex*_) is calculated using the following equation [14, 18]:

(9) $$\begin{array}{c} H_{ex}=\frac{A_{U}}{370}+\frac{A_{Th}}{259}+\frac{A_{K}}{4810}\leq 1. \end{array}$$

#### 2.5.6. Internal Hazard Index (*H*_in_)

The internal hazard index is an expression of internal exposure that comes from radon and its short-lived progeny. The internal hazard index (*H*_*in*_) is also calculated as follows [14, 18]:

(10) $$\begin{array}{c} H_{in}=\frac{A_{U}}{185}+\frac{A_{Th}}{259}+\frac{A_{K}}{4810}\leq 1, \end{array}$$

where the expression  *A*_*U*_, *A*_*Th*_, and *A*_*K*_ found in the above expressions represent the activity concentrations of uranium (^238^U), thorium (^232^Th), and potassium (^40^K) in Bq/kg, respectively.

#### 2.5.7. Excessive Lifetime Cancer Risk (ELCR)

This is associated with the probability of developing cancer over a lifetime at a given exposure level. An increase in the ELCR causes a proportionate increase in the rate at which an individual can get cancer of blood cancer, breast cancer, and prostate cancer [19]. The excessive lifetime cancer risk is calculated as follows [20]:

(11) $$\begin{array}{c} \text{ELCR}=\text{AEDE}\times \text{DL}\times \text{RF}. \end{array}$$

AEDE stands for annual effective dose equivalent, and DL for average life expectancy which is an average of 70 years. RF factor is cancer risk per sievert (Sv^−1^) from the ICRP [21], and its value is 0.05 Sv^−1^ and is used for the public given by [20]. It is a value depicting the number of cancers expected in each number of people on exposure to a carcinogen at a given dose. The threshold value of ELCR is 0.29 × 10^−3^.

## 3. Results and Discussion

### 3.1. Radioactivity Concentrations and Radiological Hazard Indices

**Figure 2:**
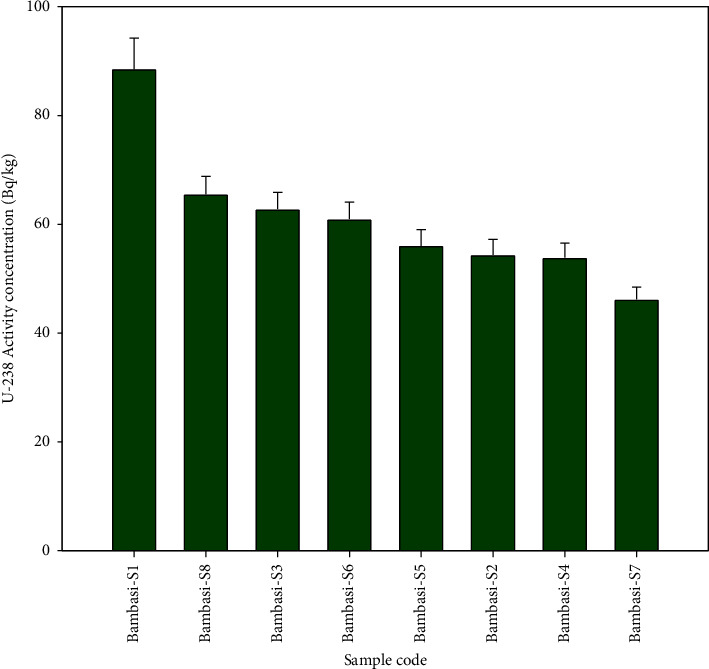
Specific activity concentration of ^238^U radionuclide within the soil samples.

**Figure 3:**
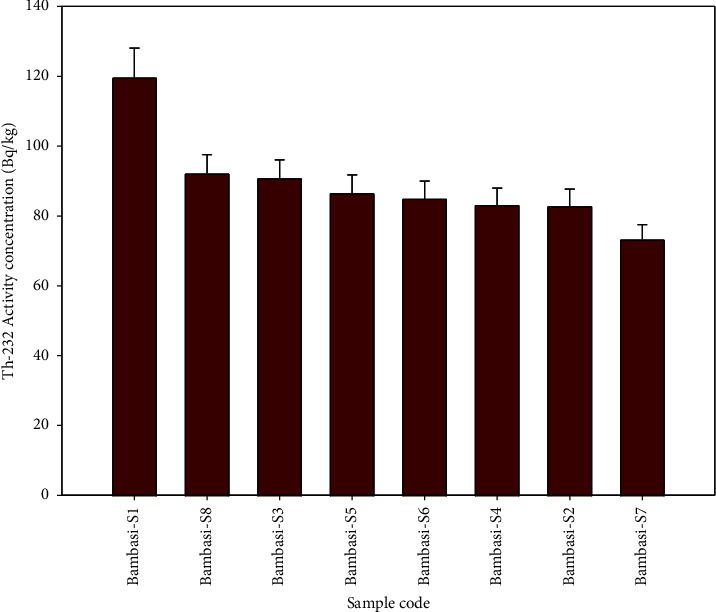
Specific activity concentration of ^232^Th radionuclide within the soil samples.

**Figure 4:**
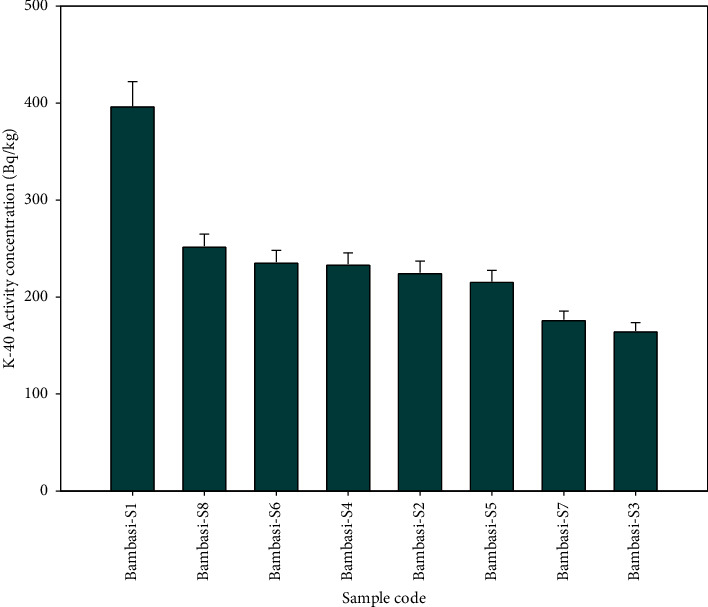
Specific activity concentration of ^40^K radionuclide within the soil samples.

The Bambasi-S1 soil exhibits the highest radioactivity concentration of ^238^U, while the lowest concentration is observed in Bambasi-S7 soil (see Figure 2).  Figure 3 shows that a higher concentration of ^232^Th found in Bambasi-S1 soil, with the lowest concentration found in Bambasi-S7 soil. As shown in Figure 4, Bambasi-S1 soil registers the highest concentration of ^40^K, while the lowest concentration is observed in Bambasi-S3 soil. The average radioactivity concentrations for all soil samples are 61 ± 3.2 Bq/kg for ^238^U, 89.2 ± 5.3 Bq/kg for ^232^Th, and 237.7 ± 12.7 Bq/kg for ^40^K. From the result values, as shown in Table 2, the mean activity concentration of ^238^U and ^232^Th in the soil of the Bambasi district surpassed the recommended global population weighted average values of 32 and 45 Bq/kg, respectively. It is known that the recommended global population weighted average values of ^238^U, ^232^Th, and ^40^K in soil are 32, 45, and 420 Bq/kg, respectively [1].

The values of radium equivalent (Ra_eq_) for all soil samples varied from 102.39 ± 11.86 to 289.9 ± 8.8 Bq/kg, with a mean value of 192.5 ± 11.2 Bq/kg (Table 2). The mean value of Ra_eq_ is below 370 Bq/kg, which is the recommended safe value for radium equivalent.

**Figure 5:**
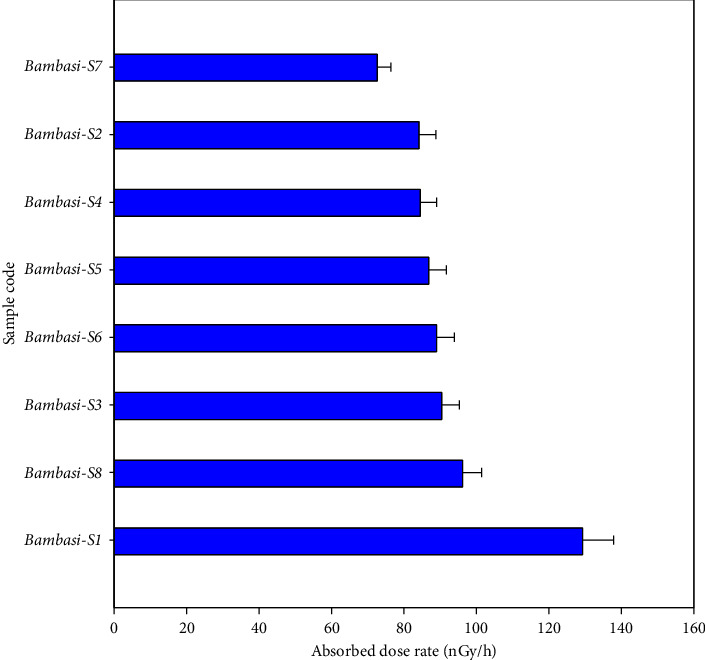
The absorbed dose rate values of the soil samples.

**Figure 6:**
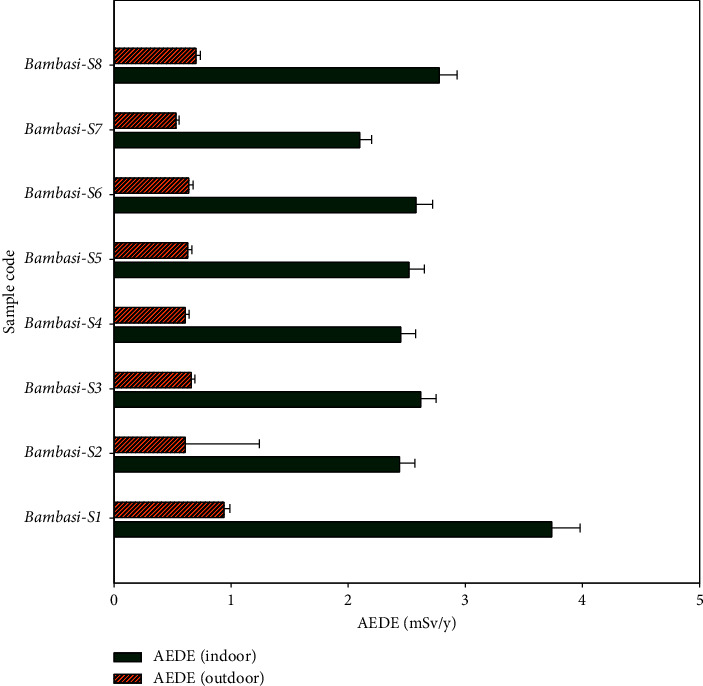
The comparison of indoor and outdoor annual effective dose equivalent values.

Table 3 shows the absorbed dose rate for all soil samples ranged from 72.7 ± 3.7 to 129.3 ± 8.6 nGy/h, with an average value of 91.6 ± 5.1 nGy/h, surpassed the recommended safe value of 59 nGy/h (Figure 5). The annual effective dose rate values for indoor exposure ranged from 2.10 ± 0.10 to 3.74 ± 0.24 mSv/y, with average value of 2.65 ± 0.14 mSv/y, while for outdoor exposure, they varied from 0.53 ± 0.026 to 0.94 ± 0.05 mSv/y, with an average of 0.66 ± 0.1 mSv/y. According to Table 3, the annual effective dose rate for all soil samples exceeds the recommended mean external radiation safe value of 0.48 mSv/y. Values of the indoor annual effective dose rates are higher than those of outdoor rates, as shown in Figure 6. However, the mean annual effective dose rate remains below the global average annual effective safety threshold of 1 mSv/y.

**Figure 7:**
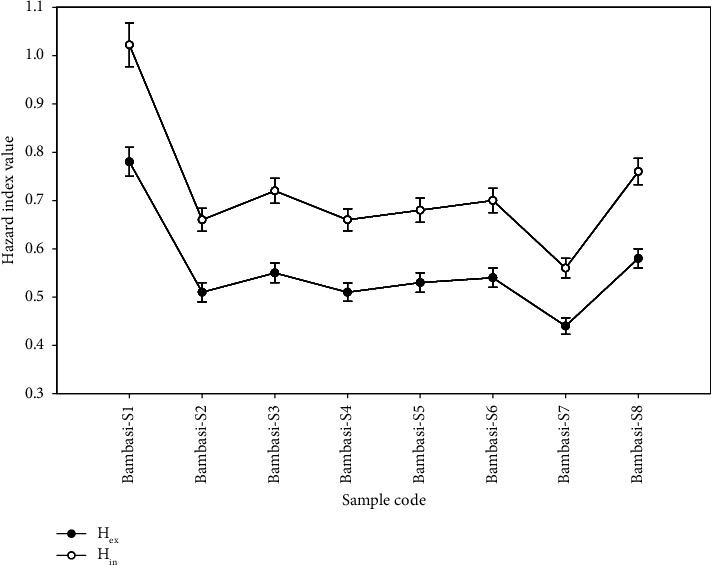
The comparison of internal and external hazard index values of the soil samples.

In Table 4, the external hazard indexes (*H*_ex_) and internal hazard index (*H*_in_) for all soil samples, not include Bambasi-S1 sample, are less than 1. Their values ranged from 0.44 ± 0.017 to 0.78 ± 0.03 for H_ex_, with an average of 0.55 ± 0.02, and from 0.56 ± 0.02 to 1.022 ± 0.045 for *H*_in_, with an average of 0.72 ± 0.05. The mean values for both external and internal hazard indexes fall below the recommended safe limit of one (Figure 7).

As shown in Figure 6, the values of the indoor annual effective dose rate signify a substantial annual radiation exposure for human tissues. Therefore, implementing safety measures for individuals residing in this environment is advisable. Additionally, Figure 7 shown that the values of the internal hazard indexes exceed those of the external hazard indexes. The gamma index values ranged from 0.57 ± 0.022 to 1.02 ± 0.04, with an average of 0.72 ± 0.02, which is below the recommended safe value of one. The mean values for the gamma index (*I*_*γ*_), internal hazard index (*H*_in_), and external hazard index (*H*_ex_) for all soil samples fall below the safe threshold of one. This indicates that the levels of radon and its short-lived daughters in the soils do not pose significant health risks to the respiratory organs of individuals living in the Bambasi district. However, the excessive lifetime cancer risk (ELCR) values ranged from (1.85 ± 0.09) × 10^−3^ to (3.29 ± 0.175) × 10^−3^, with a mean value of (2.3 ± 0.6) × 10^−3^, surpassed the threshold value of 0.29 × 10^−3^ (Table 4).

### 3.2. Comparison of Activity Concentration with Similar Studies

**Figure 8:**
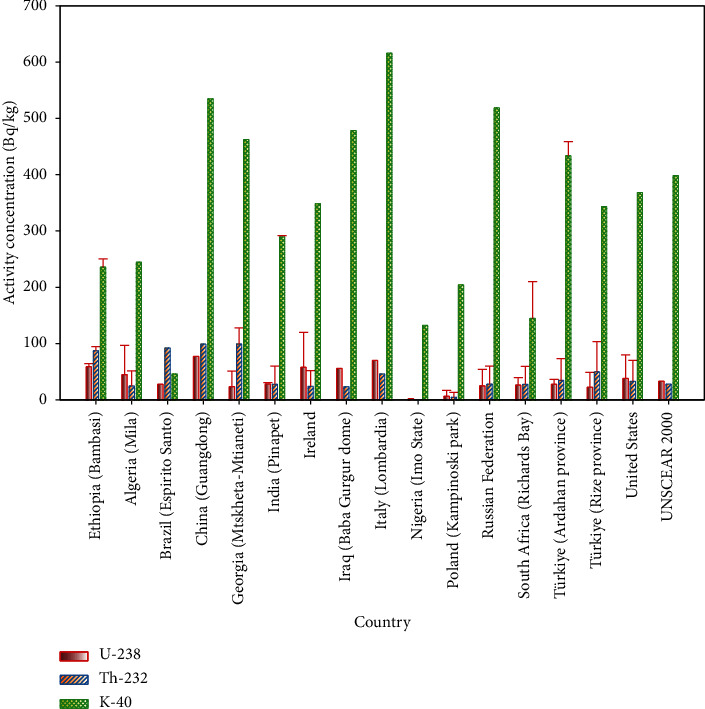
The comparison of radioactivity concentration with different countries province soils.

As shown in Table 5, a comparison was made between the radioactivity concentration of soils in the Bambasi district and similar studies conducted in other countries. The results reveal that Bambasi soils exhibit an average activity concentration of 61 ± 3.2 Bq/kg for ^238^U, 89.2 ± 5.3 Bq/kg for ^232^Th, and 237.7 ± 12.7 Bq/kg for ^40^K. The mean activity concentrations of ^238^U, ^232^Th, and ^40^K in Bambasi soils are lower than those observed in soils from the Guangdong province of China [24] and Lombardia region of Italy [28], as shown in Figure 8. However, when compared to similar studies, as shown in Figure 8, the measured mean activity concentrations of ^238^U, ^232^Th, and ^40^K in Bambasi soils are higher than those in soils from the Kedah region of Malaysia [13], Mila region of Algeria [22], Panipet region of India [26], Baba Gurgur dome of Kirkuk oil field in Iraq [27], Imo state of Nigeria [29], Kampinos park of Poland [30], and Richard Bay of South Africa [31]. Also, the measured radioactivity concentrations found in Bambasi district soil shows that it have higher concentrations than Assosa City soil, which is the capital city of Benishangul Gumuz region. Assosa City soil has a mean activity concentration of 45.2 ± 2.3, 70 ± 3.8, and 238.8 ± 11.6 Bq/kg for ^238^U, ^232^Th, and ^40^K, respectively [34]. This research provides valuable insights for future investigations due to its findings of elevated radioactivity concentrations, which are higher than international safety standards set by UNSCEAR. Numerous studies in the past have explored various aspects related to radionuclide concentration in soil, investigations into the transfer of radioactive nuclides to vegetation plants from the soil, external exposures, deposition and distribution of radionuclides in minerals and rocks, as well as radionuclides in medicinal plants, rocks as sources of raw materials for building materials, and environmental assessments [35–42].

### 3.3. Statistical Analysis

In this study, the analysis of the statistical data was performed using an Excel workbook sheet. The statistical correlation among ^238^U, ^232^Th, ^40^K, and radiological dose index values was examined using a Quantile-Quantile (Q-Q) plot, frequency distribution, and Pearson correlation (Table 6).

#### 3.3.1. Q-Q Plot and Frequency Distribution

**Figure 9:**
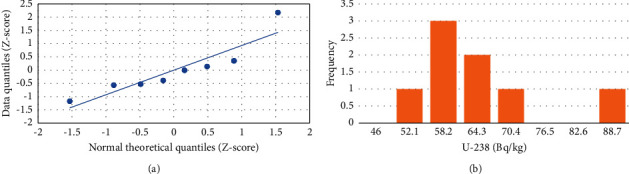
Plots for ^238^U. (a) Quantile-quantile and (b) frequency distribution.

**Figure 10:**
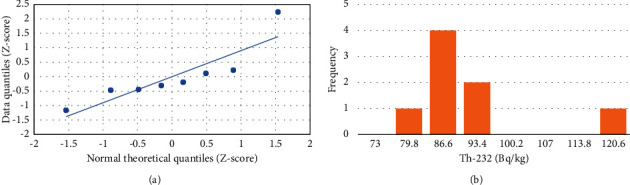
Plots for ^232^Th. (a) Quantile-quantile and (b) frequency distribution.

**Figure 11:**
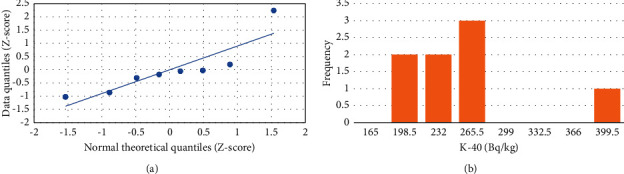
Plots for ^40^K. (a) Quantile-quantile and (b) frequency distribution.

*The* Q-Q plot uses as indicator of whether the linearity of the points or data points lies on the axis, which is related to normal distribution. When data points follow nonlinear, suggesting that the data are not normal distribution. The frequency distributions illustrate the frequency of occurrences for each possible value in a dataset. As shown in Figures 9(a), 10(a), and 11(a), the Q-Q plots suggest that the concentrations follow a normal distribution while the frequency distributions for ^238^U and ^232^Th activity concentrations exhibit multimodality, as shown in Figures 9(b) and 10(b). The activity concentration of ^40^K indicates a normal distribution (Figure 11(b)).

#### 3.3.2. Pearson's Correlation Coefficient Analysis

One of the statistical analysis methods that measures the strength and direction of a linear relationship between two variables is the Pearson correlation coefficient (PCC). By using this method, the degree of association among the measured radiological parameters was determined. The PCC result value *r* is found in the range of −1 to 1 [27, 43]. Using the Pearson correlation matrix, the correlation between ^238^U and ^232^Th, ^238^U and ^40^K, and ^232^Th and ^40^K, along with their correlations with hazard indexes of *D*, Ra_eq_, and AEDR, was estimated.

**Figure 12:**
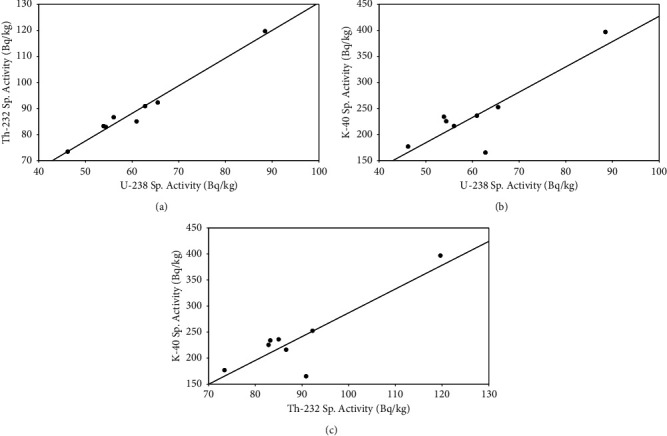
The Pearson correlation between (a) ^238^U and ^232^Th, (b) ^238^U and ^40^K, and (c) ^232^Th and ^40^K.

The Pearson correlation result for ^238^U and ^232^Th is close to one, which is *r* *=* 0.98, indicate that the two radioactive nuclides have strongly positive correlations (Figure 12(a)). For ^238^U and ^40^K, the Pearson correlation value is 0.86, indicate a strong positive relation (Figure 12(b)). As shown in Figure 12(c), for ^226^Th and ^40^K, the correlated value is *r* = 0.87, indicate a strong positive direction relation. In general, the radionuclides of the ^238^U, ^232^Th, and ^40^K decay series exhibit strong positive proportional correlation over the given decay time.

**Figure 13:**
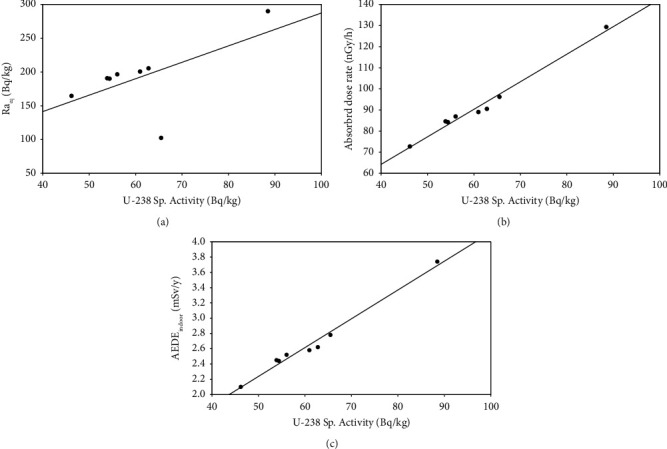
The Pearson correlation between (a) ^238^U and radium equivalent (Ra_eq_), (b) ^238^U and absorbed dose rate (D), and (c) ^238^U and indoor annual effective dose equivalent (AEDE).

As shown in Figures 12(a), 12(b), and 12(c), the Pearson correlation plots have shown a normal distribution of ^238^U, ^232^Th, and ^40^K in Bambasi soil. Their quantities by concentration are directly proportional, especially for ^238^U and ^232^Th at soil collected sites in the Bambasi district. The correlated values between uranium (^238^U) with radium equivalent (Ra_eq_), absorbed dose rate (D), and annual effective dose equivalent (AEDE) are 0.59, 0.99, and 0.99, respectively, indicate a strong positive directional correlations (Figures 13(a), 13(b), and 13(c)).

## 4. Conclusions

The mean activity concentration of ^238^U and ^232^Th and the absorbed dose rate values of all soil samples exceed their respected safe values; therefore, the natural radioactivity concentrations found in Bambasi district soils need further investigations. Also, the indoor annual effective dose rate values critically surpassed the recommended world annual average safe values. From the measured values, natural radioactivity concentration level of ^238^U and ^232^Th in all soil samples exceeds the recommended safe values, and precautions or safety measures are necessary around Bambasi district to avoid significant health problems on the community. From comparison of the radioactivity levels of Bambasi district soil samples with similar studies from different countries provinces, it can be concluded that continuous monitoring is necessary for Bambasi district soil.

## Figures and Tables

**Table 1:** Sample collected locations in Bambasi town using global positioning system (GPS).

No.	Sample code	Latitude (North)	Longitude (East)
1	Bambasi-S1	9.751911°	34.726703°
2	Bambasi-S2	9.751736°	34.729544°
3	Bambasi-S3	9.753097°	34.73175°
4	Bambasi-S4	9.756256°	34.730528°
5	Bambasi-S5	9.757667°	34.728025°
6	Bambasi-S6	9.760117°	34.726661°
7	Bambasi-S7	9.761314°	34.728581°
8	Bambasi-S8	9.759558°	34.731228°

**Table 2:** Mean radioactivity concentrations in the collected soil samples.

No.	Sample code	*A*_*U*_ (Bq/kg)	*A*_*Th*_ (Bq/kg)	*A*_*K*_ (Bq/kg)	Ra_eq_ (Bq/kg)
1	Bambasi-S1	88.49 ± 5.73	119.65 ± 8.45	396.71 ± 25.39	289.9 ± 8.8
2	Bambasi-S2	54.39 ± 2.85	82.83 ± 4.91	225.27 ± 11.73	190.09 ± 0.75
3	Bambasi-S3	62.78 ± 3.09	90.87 ± 5.17	165.12 ± 8.29	205.38 ± 11.02
4	Bambasi-S4	53.88 ± 2.68	83.2 ± 4.76	233.92 ± 11.58	190.7 ± 10.37
5	Bambasi-S5	56.05 ± 2.99	86.6 ± 5.2	216.08 ± 11.6	196.5 ± 11.28
6	Bambasi-S6	60.95 ± 3.14	85 ± 5.01	235.93 ± 12.13	200.6 ± 11.17
7	Bambasi-S7	46.2 ± 2.25	73.4 ± 4.12	176.78 ± 8.63	164.6 ± 15
8	Bambasi-S8	65.49 ± 3.30	92.25 ± 5.33	252.39 ± 12.54	102.39 ± 11.86
	Mean value	61 ± 3.2	89.2 ± 5.3	237.7 ± 12.7	192.5 ± 11.2

**Table 3:** Results of radiation hazard indexes for the collected soil samples.

No.	Sample code	*D* (nGy/h)	*E* (mSv/y)_Indoor_	*E* (mSv/y)_Outdoor_	*I*_*γ*_
1	Bambasi-S1	129.3 ± 8.6	3.74 ± 0.24	0.94 ± 0.05	1.02 ± 0.04
2	Bambasi-S2	84.2 ± 4.6	2.44 ± 0.13	0.61 ± 0.63	0.67 ± 0.02
3	Bambasi-S3	90.5 ± 4.8	2.62 ± 0.13	0.66 ± 0.03	0.71 ± 0.02
4	Bambasi-S4	84.58 ± 4.4	2.45 ± 0.127	0.61 ± 0.032	0.67 ± 0.025
5	Bambasi-S5	86.9 ± 4.8	2.52 ± 0.13	0.63 ± 0.034	0.69 ± 0.028
6	Bambasi-S6	89 ± 4.9	2.58 ± 0.14	0.64 ± 0.035	0.70 ± 0.027
7	Bambasi-S7	72.7 ± 3.7	2.10 ± 0.10	0.53 ± 0.026	0.57 ± 0.022
8	Bambasi-S8	96.2 ± 5.22	2.78 ± 0.15	0.70 ± 0.037	0.76 ± 0.029
	Mean value	91.6 ± 5.1	2.65 ± 0.14	0.66 ± 0.1	0.72 ± 0.02

**Table 4:** The external hazard index, internal hazard indexes, and excessive lifetime cancer risk values of the collected soil samples.

No.	Sample code	*H*_ex_	*H*_in_	ELCR (×10^−3^)
1	Bambasi-S1	0.78 ± 0.03	1.022 ± 0.045	3.29 ± 0.175
2	Bambasi-S2	0.51 ± 0.02	0.66 ± 0.024	2.13 ± 2.2
3	Bambasi-S3	0.55 ± 0.02	0.72 ± 0.026	2.31 ± 0.105
4	Bambasi-S4	0.51 ± 0.019	0.66 ± 0.23	2.13 ± 2.2
5	Bambasi-S5	0.53 ± 0.02	0.68 ± 0.025	2.205 ± 0.119
6	Bambasi-S6	0.54 ± 0.02	0.70 ± 0.025	2.24 ± 0.12
7	Bambasi-S7	0.44 ± 0.017	0.56 ± 0.02	1.85 ± 0.09
8	Bambasi-S8	0.58 ± 0.02	0.76 ± 0.027	2.45 ± 0.12
	Mean value	0.55 ± 0.02	0.72 ± 0.05	2.3 ± 0.6

**Table 5:** Comparison of the mean activity concentration of ^238^U,^232^Th, and^40^K within Bambasi soils in Ethiopia with other countries' province soils.

No.	Country	Average radioactivity concentrations (Bq/kg)			Reference
		^238^U/^226^Ra	^232^Th	^40^K	
1	Ethiopia (Bambasi)	61 ± 3.2	89.2 ± 5.3	237.7 ± 12.7	Present study
2	Algeria (Mila)	46.7	26.7	246.5	[22]
3	Brazil (Espirito Santo)	30	94	48	[23]
4	China (Guangdong)	79.3	101	535.8	[24]
5	Georgia (Mtskheta-Mtianeti)	25.4	26.9	464	[25]
6	India (Pinapet)	30.24 ± 0.53	29.89 ± 0.61	291.06 ± 0.57	[26]
7	Ireland	60	26	350	[1]
8	Iraq (Baba Gurgur dome)	57.8	25.4	479.9	[27]
9	Italy (Lombardia)	72	48	617	[28]
10	Nigeria (Imo state)	4.15	1.64	134.13	[29]
11	Poland (Kampinoski park)	8.54	6.65	206	[30]
12	Russian Federation	27	30	520	[1]
13	South Africa (Richards Bay)	28.26 ± 11.40	29.64 ± 11.50	146.77 ± 63.30	[31]
14	Türkiye (Ardahan province)	29.9 ± 6.2	36.7 ± 6.8	435.1 ± 23.9	[32]
15	Türkiye (Rize province)	24.5	51.8	344.9	[33]
16	United States	40	35	370	[1]
17	UNSCEAR value	32	45	420	[1]

**Table 6:** Pearson correlation matrix values between ^238^U, ^232^Th, and ^40^K, radium equivalent, absorbed dose rate, and indoor annual effective dose rate of the Bambasi soil samples.

	^238^U	^232^Th	^40^K	Ra_eq_	*D*	AEDE
^238^U	1	0.98	0.86	0.59	0.99	0.99
^232^Th		1	0.87	0.63	0.99	0.99
^40^K			1	0.58	0.91	0.91
Ra_eq_				1	0.626	0.629
D					1	0.99
AEDE						1

## Data Availability

Data are available upon reasonable request to the corresponding author.
